# Pneumothorax Following ERCP: Report of Four Cases and Review of the Literature

**DOI:** 10.1007/s10620-012-2150-3

**Published:** 2012-03-31

**Authors:** Nicolien J. Schepers, Henk R. van Buuren

**Affiliations:** Department of Gastroenterology and Hepatology, Erasmus University Medical Centre, Room Ha-203, PO Box 2040, 3000 CA Rotterdam, The Netherlands

**Keywords:** ERCP, Sphincterotomy, Pneumothorax, Complication

## Abstract

We report four patients with pneumothorax as a complication of ERCP with sphincterotomy. With conservative treatment all patients recovered. Previously, 16 comparable cases have been reported in the literature. The main risk factor for this rare complication seems (pre-cut) sphincterotomy. Pneumothorax is usually right-sided or bilateral and accompanied by pneumomediastinum, pneumoretroperitoneum and subcutaneous emphysema. The prognosis seems favourable with a non-surgical approach including intravenous antibiotics, fasting and when indicated chest tube drainage.

## Introduction

Endoscopic retrograde cholangiopancreatography (ERCP) is a complex, technically demanding procedure with a considerable potential for serious complications. The rate of specific complications—including pancreatitis, bleeding, sepsis and perforation—reportedly ranges from 5 to 6.9 %, with a mortality rate of 0.33 % [1, 2].

One of the most feared complications is perforation. The most frequent type is retroperitoneal perforation. This usually occurs after sphincterotomy, with a reported incidence of 0.1–1 % [3–5]. A dramatic complication of retroperitoneal perforation is the development of pneumothorax. As this is a rare, unexpected, frightening and potentially life-threatening event, all those involved in the care of patients undergoing ERCP should be aware of this potential complication and have knowledge of the aetiology, therapeutic principles and prognosis. We here report four patients with post-sphincterotomy pneumothorax who were observed over a 16-year period in a university hospital. In addition, a short review of previously reported cases is presented.

## Case Series

### Case 1

**Fig. 1:**
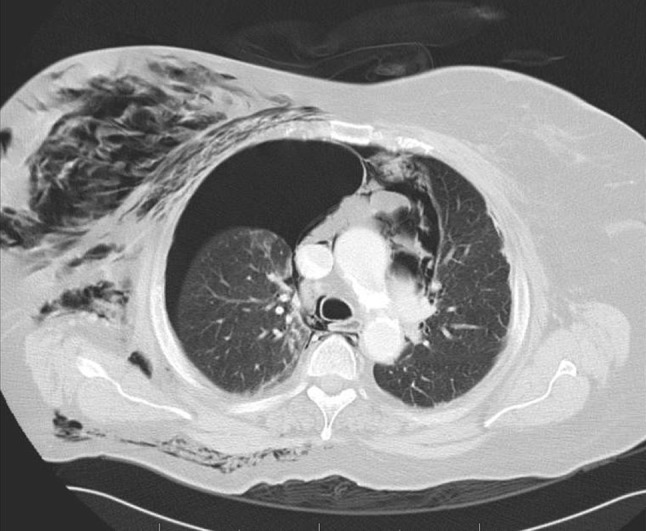
*Case 1* Thoracic CT showing right-sided tension pneumothorax with mediastinal shift to the left side. There is a smaller right-sided pneumothorax and also presence of mediastinal, soft-tissue and subcutaneous air

**Fig. 2:**
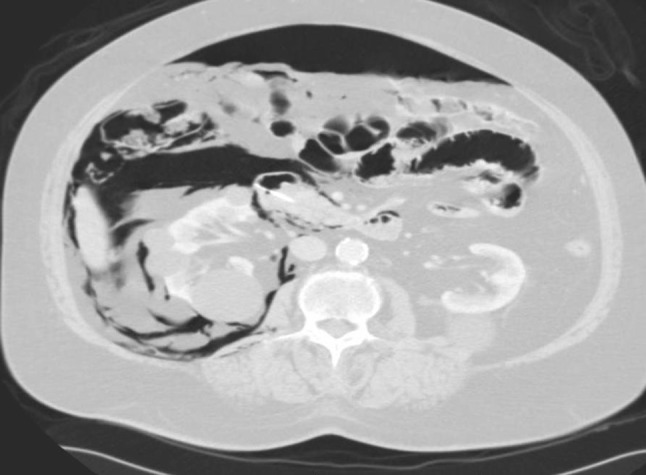
*Case 1* Abdominal CT-scan showing intra- and retroperitoneal free air

**Fig. 3:**
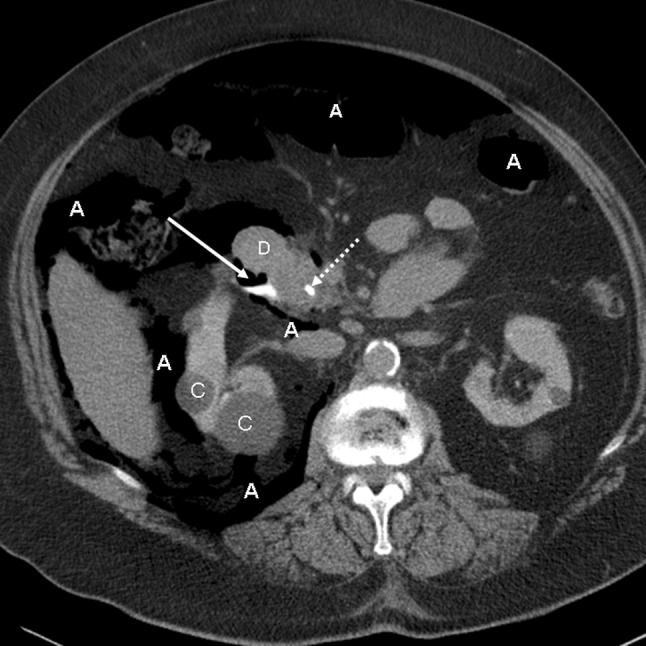
*Case 1* Abdominal CT-scan with oral contrast administration. At the site of the papilla of Vater there is minimal extravasation of contrast indicating perforation (*solid arrow*). *Dotted arrow* biliary endoprosthesis in duodenum, *A* intra- and retroperitoneal air, *C* renal cortical cysts, *D* duodenum

A 76-year-old woman underwent elective endoscopic resection of a papillary adenoma. During this procedure, which included biliary sphincterotomy for removal of common bile duct stones, no particular problems were encountered. Immediately after the procedure the patient developed severe dyspnoea and massive subcutaneous emphysema of the thorax, neck, face and lower extremities. Chest X-ray and CT revealed left-sided pneumothorax, right-sided tension pneumothorax, pneumomediastinum, pneumoperitoneum and pneumoretroperitoneum (Figs. 1 and 2). CT with oral contrast showed a minimal amount of contrast leakage in the second part of the duodenum (Fig. 3). The patient was treated with oxygen, bilateral chest tube placement, antibiotics, and a nil per mouth regime. After 1 day she was transferred from the intensive care unit to the normal ward. She gradually improved and was discharged from the hospital 10 days after the procedure.

### Case 2

A 77-year-old man was admitted with obstructive jaundice. Imaging studies suggested a pancreatic head tumour. Endoscopic cholangiography showed a severe stenosis of the distal common bile duct. For selective cannulation pre-cut sphincterotomy was performed followed by placement of a plastic endoprosthesis. During this procedure retroperitoneal air was noticed. The patient became hypotensive and developed extensive subcutaneous air of the neck and head. Further imaging showed mediastinal air and bilateral pneumothorax. Conservative treatment was started, including antibiotic treatment for 5 days, intravenous fluids, nil per mouth and gastric drainage. The clinical course was uneventful and the patient made a rapid recovery within 1 week.

### Case 3

An 88-year-old woman with complaints of intermittent abdominal pain was scheduled for ERCP for removal of biliary stones. The papilla was situated in a large duodenal diverticulum. Following guidewire-assisted sphincterotomy there was immediate suspicion of an abnormal route with retroperitoneal leakage. Furthermore, there was active bleeding at the site of the sphincterotomy. Haemostasis was achieved with adrenalin injections. The patient developed gradually progressive subcutaneous emphysema, chest pain and dyspnoea. Additional imaging showed pneumomediastinum and right-sided pneumothorax. Initial treatment included insertion of a nasobiliary drain and administration of oxygen. She was transported to the intensive care unit where a chest tube was inserted and antibiotic treatment was started. The following day there was marked clinical improvement and she could return to the normal ward. The further clinical course was uneventful.

### Case 4

A 58-year-old woman underwent ERCP for further evaluation and treatment of jaundice and biliary pain. Biliary cannulation was unsuccessful and pre-cut sphincterotomy was performed. After successful cannulation, cholangiography failed to show clear biliary abnormalities. At this stage the presence of retroperitoneal and mediastinal air was noted, accompanied by right-sided pneumothorax. Conservative treatment was initiated and consisted of nil per mouth, nasobiliary drainage and antibiotics. The further clinical course was uncomplicated and the patient fully recovered.

## Summary of Reported Cases

A literature research revealed 16 additional, previously published cases of pneumothorax complicating ERCP (Table 1). It should be noted that cases with instrumental wall perforations or perforation of pre-existing duodenal ulcers were excluded. The mean age of the total reported 20 patients was 66 (range 24–89) years; 16 were female. In four cases the presence of juxtapapillary diverticula was reported and in two-thirds of cases sphincterotomy or pre-cut sphincterotomy preceded the development of pneumothorax. In 8/20 (40 %) cases pneumothorax was bilateral while in no more than one case it was only left-sided. In 2 of the 20 patients surgery was undertaken. Chest tube drainage, either uni- of bilateral, was performed in 15 (75 %) patients. In 19/20 cases the outcome was favourable, with full recovery of the patient. A fatal outcome was reported for one patient, a 78-year-old woman who presented 3 days after ERCP with sphincterotomy and failed stone extraction, and died from peritonitis and sepsis [6].

**Table 1:** Cases of pneumothorax complicating endoscopic retrograde cholangiopancreatography (ERCP)

Author	Age (years)	Gender	Indication for ERCP	ERCP procedure	Location and type of pneumothorax	Other findings^a^	Management	Outcome
Gya et al. [7]	63	F	CBD stone	Sphincterotomy	Right-sided	A, C, D	Chest tube, laparotomy	Survived
Scarlet et al. [8]	59	F	Biliary pain	Pre-cut sphincterotomy	Right-sided	B, C	Chest tube, conservative	Survived
Doerr et al. [9]	81	F	CBD stone	Failed attempt to remove CBD stone	Right-sided	A, B, D	Chest tube, conservative	Survived
Hui et al. [10]	89	F	Cholangitis, CBD stones	Failed attempt to reach papilla, B-II gastrectomy	Right-sided	–	Chest tube, conservative	Survived
Lagoudianakis et al. [11]	55	M	Cholelithiasis, jaundice	Failed attempt of catheterization papilla, sphincterotomy	Right-sided	C, D	Conservative	Survived
Markogiannakis et al. [12]	56	F	Cholangitis	Sphincterotomy, stone removal	Bilateral	A, B, C, D	Bilateral chest tube, conservative	Survived
Kocaman et al. [13]	24	M	Progressive jaundice	Brushing, endoprosthesis	Bilateral	A, B, C, D	Bilateral chest tube, laparotomy	Survived
Ferrara et al. [14]	82	M	Cholangitis, CBD stones	Sphincterotomy, stone removal	Left-sided	A, B, C, D	Chest tube, conservative	Survived
Iyilikci et al. [15]	24	F	CBD stone	Sphincterotomy, partial stone removal	Bilateral	D	Chest tube, laparotomy	Survived
Sang-Yun Song et al. [6]	78	F	CBD stone	Sphincterotomy	Right-sided tension	A, C, D	Conservative	Died
Schiavon et al. [16]	79	F	CBD stones	Sphincterotomy, Stone removal	Right-sided	A, D	Conservative	Survived
Brueck et al. [17]	39	F	CBD stones	Sphincterotomy with lithotripsy, stone removal	Bilateral	A, B, C, D	Chest tube, conservative	Survived
Fuji et al. [18]	73	F	Biliary anastomotic stricture	Balloon dilatation, endoprothesis placement	Bilateral	A, B, C, D	Bilateral chest tube, conservative	Survived
Ozgonul et al. [19]	62	F	Obstructive jaundice	Klatskin tumour, stenting	Bilateral	A, B, C, D	Bilateral chest tube, laporatomy	Survived
Sampaziotis et al. [20]	68	F	CBD stones	Extension sphincterotomy, Stone removal	Bilateral	A, B, C, D	Bilateral chest tube	Survived
Seymann et al. [21]	78	F	CBD stone	Stone removal	Bilateral	A, C, D	Bilateral chest tube	Survived
Present case 1	77	F	Resection papilla adenoma	Sphincterotomy, resection papilla adenoma	Bilateral, right-sided tension	A, B, C, D	Chest tube, conservative	Survived
Present case 2	77	M	Obstructive jaundice	Precut sphincterotomy	Bilateral	A, C, D	Conservative	Survived
Present case 3	88	F	CBD stones	Sphincterotomy, bleeding	Right-sided tension	A, C, D	Chest tube, conservative	Survived
Present case 4	58	F	Jaundice and biliary pain	Precut sphincterotomy	Right-sided?	A, C	Conservative	Survived

^a^Other findings:* A* mediastinal air,* B* intraperitoneal air,* C* retroperitoneal air,* D* subcutaneous emphysema

*CBD* common bile duct,* F* female,* M* male

## Discussion

The cumulative reported experience with pneumothorax complicating ERCP suggests that this is an exceptional complication that can occur at any age, is usually right-sided or bilateral and is typically associated with the presence of retroperitoneal, mediastinal and subcutaneous air, frequently also with intraperitoneal air. The main risk factors are (pre-cut) sphincterotomy and possibly the presence of juxtapapillary diverticula. With conservative treatment, including chest tube insertion, administration of antibiotics and a temporary nil by mouth regimen, the prognosis seems good. A notably complicated or fatal course seems uncommon.

We are aware of several unpublished cases of pneumothorax following sphincterotomy. Publishing serious complications may not be appealing, particularly when the outcome is unfavourable or fatal. This may be a significant factor contributing to underreporting of serious complications such as ERCP related pneumothorax. Although the incidence of this complication is without doubt low, we speculate that the true occurrence may be higher, and the course more often complicated than is suggested by currently available data.

### Pathophysiology

Several pathophysiological mechanisms underlying ERCP related pneumothorax have been proposed. The most likely route is that air enters the retroperitoneal space after interruption of the duodenal barrier. The most frequent cause is (pre-cut) sphincterotomy with false direction or an incision that is too large or deep. In many cases altered or variant anatomy, secondary to tumours, diverticula or other causes, is likely to be of importance. Insufflation of air may contribute to relatively fast accumulation of air in the retroperitoneum. Subsequently air can spread to the mediastinum, the subcutaneous tissues and also to the peritoneal cavity. Finally, mediastinal air can gain access to the pleural cavity, possibly due to rupture of the parietal pleura. Depending on the amount of air this can give rise to pneumothorax of variable severity. It has been suggested that a continuum of fascial planes connects cervical soft tissues with the mediastinum and retroperitoneum [22]. This particular spatial anatomy could facilitate rapid movement of air from one compartment to another.

Several observations indicate that a complete transmural defect is not a prerequisite for developing pneumoretroperitoneum and its sequelae [23–25]. Thus local weakening of the duodenal-retroperitoneal barrier, for instance after sphincterotomy or balloon dilatation, or secondary to tumour or inflammation, may occasionally allow air to enter the retroperitoneum. This is supported by several reported cases with pneumoretroperitoneum and pneumothorax unrelated with (pre-cut) sphincterotomy (Table 1).

An alternative pathway is that pores in the diaphragm, formed either congenitally or acquired, may allow air to move between the abdominal and thoracic cavity. Porous diaphragm syndromes are characterized by the passages of fluids, gases, tissues, secretions and intestinal content through diaphragmatic pores from the peritoneal cavity into the ipsilateral hemithoracic space [26]. This is also the pathophysiological mechanism underlying hydrothorax associated with peritoneal dialysis and ascites. The fact that intraperitoneal air is certainly not an uniform finding in patients with ERCP associated pneumothorax does not support this as a central mechanism. It has also been hypothesized that alveolar rupture may occur due to increased intrathoracic pressure, particularly in patients poorly tolerating endoscopic procedures [20]. The Vasalva manoeuvre has been strongly associated with development of subcutaneous emphysema, pneumomediastinum, and more rarely pneumothorax [27].

Studies employing standard CT after sphincterotomy indicate that air in the retroperitoneal space, indicative for duodenal perforation, can be found in up to 29 % of cases, suggesting that in the large majority of cases this is a sub-clinical problem not requiring specific therapy [28, 29].

Juxtapapillary diverticula are a possible risk factor for retroperitoneal perforation [30, 31] and were described in 20 % of cases in this review. However, the importance of diverticula as an independent risk factor is uncertain as the prevalence in populations undergoing ERCP may be as high as 20–27 % [32, 33].

### Diagnosis

Depending on the amount of leakage, retroperitoneal perforation and pneumothorax may become manifest either during (3 of our 4 cases) or after ERCP. This can be as fluoroscopically or radiologically visible accumulation of retroperitoneal, mediastinal or intrapleural air or by symptoms such as unrest, tachycardia, dyspnoea, subcutaneous emphysema or hypoxemia. Also, contrast can be visible in the retroperitoneal space. Retroperitoneal perforation is usually not visible endoscopically. Changes in vital signs, dyspnoea and decreasing oxygen saturation, especially following (pre-cut) sphincterotomy, should lead to considering possible retroperitoneal perforation or pneumothorax. Imaging other than a chest and a plain abdominal X-ray is probably not necessary in the majority of cases with retroperitoneal air and pneumothorax. When this is not evident and a patient after ERCP deteriorates clinically, with symptoms and signs suggesting a possible serious complication, abdominal and/or chest CT is indicated [34]. In many of the reported cases of pneumothorax after ERCP, even extensive radiographic imaging studies failed to show the site of perforation [6, 7, 12–14, 16–18, 21]. Laparotomy may fail to show perforation [13, 15] even when this was radiologically shown [7]. More important, however, is the clear absence of therapeutic implications of documenting actual intestinal leakage or the exact site of perforation. Therefore diagnostic studies aimed at documenting leakage seem usually not indicated or useful. Obviously the situation is different when there is evidence to suggest that instrumental trauma may have caused bowel wall perforation. This may be a particular consideration in the presence of strictures, external tumour or inflammatory compression and altered anatomy of the upper gastrointestinal tract, such as after gastric surgery, especially when problems were encountered during the endoscopy.

### Treatment

Obviously, there are no large series or controlled studies with respect to the optimal treatment of this complication. Comparable with the usual management of retroperitoneal duodenal perforation [35–37], the experience here reported with ERCP related pneumothorax indicates that a non-surgical approach can be followed. The conservative treatment regimen employed by most authors included the administration of oxygen and of intravenous antibiotics, a variable period of fasting (nil by mouth) and if indicated, with respect to the severity of pneumothorax and hypoxemia, uni- or bilateral pleural drainage. Patients should be carefully observed and any concern about the clinical evolution should lead to consider additional imaging and surgical consultation. When the initial conservative treatment strategy of retroperitoneal perforation is not successful surgical closure of the leak has been found feasible and effective [38].

Duodenal perforation secondary to sphincterotomy has been treated with endoscopic clips [39, 40]. In two of our patients in whom pneumothoraces were detected during ERCP, nasobiliary drains were placed in order to divert bile away from the site of perforation. Given the reported favourable outcome in many cases not treated with clips or biliary drainage, it is uncertain whether these procedures can be considered an important part of management. The same may apply to the necessity of gastric drainage.

### Conclusion

Pneumothorax is a rare but frightening complication of ERCP. The most common pathophysiology is retroperitoneal perforation due to (pre-cut) sphincterotomy, followed by development of pneumomediastinum and pneumothorax. With conservative treatment, including pleural drainage when indicated, rapid and complete recovery can be expected.

## Abbreviations

CT: Computed tomography
ERCP: Endoscopic retrograde cholangiopancreatography
